# Objective and Subjective Stress Parameters in Response to High and Low-Fidelity Simulation Activities

**DOI:** 10.3390/ijerph19052980

**Published:** 2022-03-03

**Authors:** Marta Czekirda, Patrycja Misztal-Okońska, Anna Włoszczak-Szubzda, Mariusz Goniewicz, Mateusz Cybulski, Krystyna Kowalczuk, Noemi Jaszyna, Maria Pyć, Mariusz Gnat, Joanna Girzelska, Ewa Guz, Mariusz Sutryk, Wioletta Tuszyńska-Bogucka, Krzysztof Goniewicz, Ahmed M. Al-Wathinani, Amir Khorram-Manesh

**Affiliations:** 1 Faculty of Human Sciences, University of Economics and Innovation, 20-209 Lublin, Poland; marta-czekirda@wp.pl (M.C.); noemi.czekirda@wsei.lublin.pl (N.J.); maria.pyc@wsei.lublin.pl (M.P.); mariusz.gnat@wsei.lublin.pl (M.G.); joanna.girzelska@wsei.lublin.pl (J.G.); ewa.guz@wsei.lublin.pl (E.G.); mariusz.sutryk@wsei.lublin.pl (M.S.); wioletta.tuszynska-bogucka@wsei.lublin.pl (W.T.-B.); 2 Department of Emergency Medicine, Faculty of Medical Sciences, Medical University of Lublin, 20-093 Lublin, Poland; patrycja.okonska@umlub.pl (P.M.-O.); mariusz.goniewicz@umlub.pl (M.G.); 3 Department of Integrated Medical Care, Faculty of Health Sciences, Medical University of Białystok, 15-096 Białystok, Poland; mateusz.cybulski@umb.edu.pl (M.C.); krystyna.kowalczuk@umb.edu.pl (K.K.); 4 Department of Aviation Security, Military University of Aviation, 08-521 Dęblin, Poland; k.goniewicz@law.mil.pl; 5 Department of Emergency Medical Services, Prince Sultan Bin Abdulaziz College Emergency Medical Services, King Saud University, Riyadh 11451, Saudi Arabia; ahmalotaibi@ksu.edu.sa; 6 Department of Surgery, Institute of Clinical Sciences, Sahlgrenska Academy, Gothenburg University, 41345 Gothenburg, Sweden; amir.khorram-manesh@surgery.gu.se; 7 Gothenburg Emergency Medicine Research Group (GEMREG), Sahlgrenska Academy, Gothenburg University, 41305 Gothenburg, Sweden

**Keywords:** stress, stressors, cortisol, medical simulation, low-fidelity simulation, high-fidelity simulation, stress appraisal questionnaire, KOS-B, education, nursing

## Abstract

Nursing graduates are required to have both excellent theoretical and practical skills that should be used during stressful emergency interventions. Since the received knowledge should be practiced to gain skills and trained to achieve competences, simulation exercises can be beneficial to even reduce the stress that each individual may face during emergency management of patients. A total of 146 first-year nursing students participated in the study, including 124 women and 22 men aged between 19 and 50 years, with a mean age of 32 years. The objective method estimated psychophysiological parameters (serum cortisol). Objective and subjective methods were used. The subjective method assessed stress experienced by students based on the standardized Stress Appraisal Questionnaire Version B for dispositional assessment. The study was conducted in the Monoprofile Medical Simulation Centre at the University of Economics and Innovation in Lublin, Poland and was approved by the University Research Ethics Committee. Both participants under and over 25 years of age showed increased levels of stress after low and high-fidelity simulations, with statistically significantly higher stress levels found for the low fidelity method. Low-fidelity simulation methods generated a greater increase in cortisol levels, indicating a higher stress level than the high-fidelity methods. The analysis of the scores obtained in the Stress Appraisal Questionnaire (KOS-B) showed that higher cortisol levels after the low-fidelity simulation reduced the subjective perception of a threat, while higher cortisol levels before the high-fidelity simulation promoted higher intellectual activity among the students. Levels of stress in the education of nursing students using low and high-fidelity methods can limit the sense of threat and activate professional task performance. The use of low and high-fidelity simulation does not generate destructive stress levels.

## 1. Introduction

The use of modern technologies has become a standard in the educational process. In the case of medical universities, this approach guarantees the development of skills, competences and professionalism in students. A special role is played by simulation-based education, which offers the possibility to recreate potential clinical cases to enable students gain experience and learn about medical procedures in a safe environment [1]. Simulators give students the opportunity to intervene and evaluate treatment and care outcomes [2]. Simulators, models and phantoms allow for the acquisition and consolidation of technical skills related to manual procedures, e.g., cardiopulmonary resuscitation, catheterization, intubation, insertion of an infusion [3]. There are many scientific reports confirming that simulation is an effective educational tool in the field of medicine, including nursing [3,4,5,6,7,8,9].

Fidelity simulation is multidimensional. The degree of realism is reflected through a detailed scenario, scenery and the selection of simulation equipment. Fidelity is also defined by the degree of accuracy and reliability of the experience. It can be low, medium, and high, and its types are physical, psychological, and conceptual. The level of fidelity should be conducive to achieving the expected educational results [10].

Low Fidelity Simulation (LFS) is performed using partial trainers i.e., mannequins or equipment representing certain anatomical parts of the human body. These are crucial instruments for learning essential nursing skills such as intramuscular or intravenous injections, inserting a peripheral IV catheter, etc. [11]. The level of fidelity should be also appropriate to the type of task and training stage. At more advanced levels of training, the level of fidelity should support higher levels of speed and practice of task [12]. The appropriate level of fidelity is dependent on the intended learning goals and cost. Different levels of fidelity may be needed for different objectives and levels of trainees [12].

High Fidelity Simulation (HFS) allows for maximum experiential learning, using phantoms with multiple complex functions that realistically reflect the vital functions of the human body. The advanced interactive capabilities of the phantoms enable students to act in real-life scenarios. Students are able to detect and correct errors themselves without negative consequences for the patient. In this way they acquire both clinical skills and social competences without a fear in a safe environment with no stress. High-fidelity simulation is performed in simulated intensive care rooms, delivery rooms, operating rooms, and even hospital emergency departments [13,14]. High-fidelity simulation has been shown to support soft skills and task-focusing, enhance the sense of self-competence, develop self-confidence, self-efficacy, sense of satisfaction, as well as clinical thinking, skills, judgment, and decision-making [11,13,15]. It also provides a safe and developmental practice space to gain experience in situations that are likely to be encountered in the future professional work [16]. The possibility to perform procedures repeatedly during practice is an excellent way to control psychological states and experiences such as uncertainty, apprehension, and anxiety that accompany the nursing profession, especially at the beginning of a career [17,18,19,20].

Previous research shows that fidelity simulation training is very popular with students and is received with enthusiasm [21,22,23,24]. Furthermore, many studies indicate that simulation based on scenarios played out by the students themselves improves teamwork skills [25,26,27].

The effectiveness of high and low-fidelity methods in the nursing education process is currently beyond question. However, there is little research that explains whether these techniques, regardless of their benefits, are also a source of stress, as in the real emergency encounter, how students cope with such situations and if this stress is beneficial. The novelty of our proposed research lies in focusing on objective indicators of stress experienced by students during high and low-fidelity simulation activities based on cortisol levels. We compared this indicator with the students’ subjective estimate of stress levels during the activities.

## 2. Aim of the Study

The aim of the study was to assess the differences in the level of stress assessed objectively (cortisol level) and subjectively (Stress Assessment Questionnaire, KOS-B), under simulation conditions using low and high-fidelity education methods.

## 3. Materials and Methods

### 3.1. Study Material

The study involved 146 students (124 females, 22 males) aged 19–50 years (M = 31.83, MD = 31.00, *SD* = 7.76) from the University of Economics and Innovation in Lublin, Poland, most of whom were professionally active (83.6%): 25 (17.1%) paramedics, 34 (23.3%) healthcare assistants, 11 (7.5%) physiotherapists, and 52 (35.6%) other healthcare professions. Secondary and higher education was reported by 83 and 63 respondents (χ^2^ = 2.740, *p* = 0.098), respectively. Detailed demographic characteristics of the study group are presented in Table 1.

### 3.2. Research Methods

The study used two research methods (a) laboratory analysis of serum cortisol levels and (b) questionnaire assessment of perceived stress (Stress Appraisal Questionnaire Version B).

Analysis of serum cortisol level: Serum cortisol was measured to objectively assess the level of stress experienced by the students. Activation of the hypothalamic-pituitary-adrenal axis in response to stressful stimuli triggers increased production of glucocorticoids to restore homeostasis. Cortisol is the main glucocorticoid hormone produced by the adrenal cortex. Its increased amounts are released in response to high levels of stress to mobilize the body’s resources. Among others, cortisol raises blood glucose levels, has immunosuppressive and anti-inflammatory effects, and is responsible for salt retention in the body. Chronically elevated cortisol may have adverse consequences for health [28,29].

### 3.3. Preparation for Blood Collection

A week before the scheduled blood collection, the students were informed how to prepare for the test. In order to obtain reliable serum cortisol measures, the students were asked to follow the following recommendations:

◦ avoid stressful situations a few days before sampling, if possible;
◦ avoid alcohol for 3 days before the analysis;
◦ report fasted for the test (12 h after the last meal);
◦ drink a glass of water a few minutes before the test;
◦ rest for at least 30 min before sampling.

Pregnant women and estrogen-treated women were excluded from the study due to elevated cortisol levels in both these groups.

### 3.4. Next Phase

The study was implemented in two stages.

- The first stage took place in December 2019 among students participating in nursing skills classes implemented in the first semester using a low-fidelity method before and after parameter measurements. Cortisol was measured in the blood sampled before and after completing measurements.
- The same procedure as in the first stage of the study was performed, i.e., blood collection took place before and after passing the parameter measurement using the high-fidelity method.

Samples were collected in the morning (between 8:00 and 10:00 a.m.) in seven groups of students and in the afternoon (between 5:00 and 6:15 p.m.) in eight groups of students. The students in each group had their blood sample taken at the same time each time (both in the first and second stage of the study), i.e., during the classes of their study group. At each stage, the students also completed The Stress Appraisal Questionnaire.

### 3.5. Laboratory Analysis—Serum Cortisol Levels

Blood was collected twice, before and after low and high-fidelity simulation sessions. A vacuum-venipuncture technique was used (by nurses) to collect blood. The tubes and referrals for the test were then coded. The blood collected from the students was centrifuged by a laboratory staff for 10 min at 3500 rpm. The obtained serum was taken to the laboratory, where it was stored at −80 °C and then analyzed.

The Alinity and Cortisol kit is a Chemiluminescent Microparticle Immunoassay (CMIA) for the quantitative determination of cortisol in human serum, plasma or urine using the Alinity analyzer. In healthy individuals, cortisol levels are regulated by a negative feedback loop in which the adrenal cortex responds to elevated adrenocorticotropic hormone (ACTH) through increasing cortisol secretion, and the pituitary gland responds to elevated cortisol by decreasing ACTH production. Plasma cortisol levels peak in the morning and decrease by about half in the evening. The analysis of the biological material was performed on the ARCHITECT i System analyzer. The results obtained in individual laboratories may differ from these data as each laboratory has its own reference range specific for a given latitude and population (we use: Laboratory of Medical Analyzes ALAB Lublin Ceramiczna ul. Ceramiczna 20-150 Lublin, Poland).

### 3.6. Reference Range for Serum Cortisol

Serum cortisol values were obtained from samples taken from adults (considered healthy) before 10:00 a.m. and after 5:00 p.m. A 95% reference range was determined for the study group in which samples were collected before and after noon. The obtained results are summarized in Table 2 below.

The objective stress assessment based on cortisol levels was performed in parallel with the subjective assessment. For this purpose, each participant was asked to complete the Stress Appraisal Questionnaire (version B). The timing of the questionnaire was in coordination with the objective assessment, i.e., before and after completing the measurements of low and high-fidelity parameters in MMSC.

### 3.7. Description of the Research Tool—KOS-B

The Stress Appraisal Questionnaire (KOS) [30] allows for the diagnosis of the type and severity of stress experienced by respondents in stressful situations. The theoretical background for the tool is the transactional model of stress and coping developed by Lazarus and Folkman [31] and the interactional model of coping based on this theory (with amendments by Wrześniewski [30]). The tool measures the appraisal of currently experienced stressful situation (‘situational stress appraisal’—KOS-A) and the related personality dispositions (‘dispositional stress appraisal’—KOS-B). The present study used the KOS-B version, which measures stress intensity in four subscales: threat—nine diagnostic items, harm/loss—four items, challenge-activity—five items, challenge-passivity—five items. The remaining statements have a buffering function. The higher the score in a given subscale, the higher the level of appraisal of a given stressful situation. The respondents are asked about how they generally perceive different stressful situations. In both parts, the responses are rated on a scale of 0 to 3, where: 0 = definitely not; 1 = rather not; 2 = rather yes, 3 = definitely yes. In practice, each part can be used separately. Completing one of the KOS parts takes about 10 min. The accuracy of the tool was determined based on factor validity and criterion (diagnostic) validity of selected subscales based on the analysis of KOS correlation coefficients with other measures of psychological properties. The reliability of the questionnaire was determined using the following methods: α-Cronbach’s internal consistency and the Guttman Split-half coefficient.

The reliability parameters of the questionnaire are satisfactory and reach the following values for the individual factors: for the situational assessment, the α-Cronbach’s for Threat = 0.90, Harm/Loss = 0.80, Challenge-activity = 0.71, Challenge-passivity = 0.76. For the dispositional appraisal, the reliability coefficient values are as follows: α-Cronbach’s for Threat = 0.90, Harm/Loss = 0.84, Challenge-activity = 0.81, Challenge-passivity = 0.79 [30].

### 3.8. Ethics

The study was conducted at the turn of 2019 and 2020 at the Monoprofile Medical Simulation Centre (MMSC). The participants were informed of the purpose of the study, as well as the possibility of withdrawing at any time and without giving any reason. All participants gave their informed and voluntary consent to participate in the study. The approval of the Bioethics Committee for Ethics operating at the Higher School of Economics and Innovation in Lublin was obtained prior to the start of the study (application No. 2019/12/1, dated 19 December 2019).

### 3.9. Statistical Analysis

Statistical analysis was performed using SPSS Statistics version 25. A level of α < 0.05 was considered statistically significant. Descriptive statistics were used to describe the variables: mean, median, standard deviation, kurtosis. Shapiro–Wilk and Kolmogorov–Smirnov tests were used to evaluate the distribution of variables. For variables significantly deviating from the normal distributions, an attempt was made to normalize by the natural logarithm. For the variables that met the assumptions for the parametric tests (measurement of cortisol after normalization), a *t*-test was used for repeated measurements. For the variables that did not meet the assumptions (KOS results) for the parametric tests, the non-parametric tests of the Wilcoxon signs test were used. Correlation tests were used to assess the dependence of quantitative variables—the Rho Spearman correlation coefficient.

## 4. Results

The Kolmogorov–Smirnov analysis of the distribution of results (for N > 100) showed that they mostly deviated from a normal distribution. Only in two cortisol measurements performed after the low-fidelity and high-fidelity simulation sessions, the distributions of the results were close to a normal distribution (Table 3).

The analysis of the mean stress scores obtained in KOS has shown the only change in the case of the general dimension (*M_KOS__GL_before_* = 22.22, *SD* = 6.07; *M__KOS_GL_after_* = 26.38, *SD* = 6.90). The mean values of the four questionnaire dimensions (threat, challenge-activity, challenge-passivity, and harm) were found to be identical before and after simulation sessions.

Psychological stress assessment based on blood sampling before the low-fidelity simulation showed that the mean cortisol level was *M__1_before_* = 90.53, with standard deviation *SD* = 39.12. After simulation, there was a statistically significant increase (*p* < 0.001) in cortisol levels to *M__1_after_* = 116.80, *SD* = 42.19. The difference in the means was 26.27.

Stress assessment based on blood sampling before the high-fidelity simulation showed that the mean cortisol level was *M__2_before_* = 112.75, with a standard deviation of *SD* = 42.44. After the high-fidelity simulation, there was an increase in cortisol level to *M__2_after_* = 117.57, *SD* = 44.80, with the difference in the means of 4.82, which did not meet the criterion of statistical significance, but remained at the trend level (*p* = 0.093), (data Table 3).

The indicators were important obtained of the level of experienced stress (blood sampling before and after low-fidelity simulation) F(1,145) = 173.170, *p* < 0.001, η2 = 0.544, the observed power of the test was 1.00. The difference in means was significant for cortisol levels (M_1_CORT_before − M_1_CORT_after = −26.27, *SD* = 24.12, t(145) = 13.159, *p* < 0.001). A negative result indicates increased cortisol levels in blood sampling “after low-fidelity the simulation”. Thus, the level of stress experienced was higher after a low-fidelity simulation session and this result was found to be consistent with the students’ subjective experience of stress (KOS low-fidelity simulation).

For the blood collection, before and after high-fidelity simulation, the difference in the means remained at a trend level (M_2_CORT_before − M_2_CORT_after = −4.82, *SD* = 34.46, t(145) = −1.691, F(1,145) =2.858, η2 = 0.019, *p* = 0.093, with the test power of 0.390).

Thus, it appears that low-fidelity simulation gave rise to higher physiological and subjectively perceived stress than high-fidelity sessions. The level of experienced stress, (criterion cortisol) and subjectively (KOS) perceived stress was higher after low-fidelity simulation sessions. For the high-fidelity simulation, the difference in the level of experienced stress (measure: cortisol and KOS) appeared to remain at the trend level.

The result calculated with α = 0.05 revealed a significant main effect for subjective stress appraisal, measured based on the total pre- and post-simulation KOS score F(1,145) = 25.548, *p* < 0.001, η2 = 0.150, with the observed power of the test of 0.999. The difference in the mean stress appraisal scores between the measurements 1 and 2 (before and after collection) proved to be significant (M__I_KOS_ − M__II_KOS_ = −4.16, *SD* = 9.95, t(145) = −5.055, *p* < 0.001).

There was a very weak statistically significant negative correlation between the sense of threat and cortisol levels after low-fidelity simulation sessions (*r* = −0.194). This indicates that higher cortisol levels after a session reduce the sense of threat (Table 4).

For the high-fidelity method, a very weak statistically significant positive correlation was found between pre-simulation cortisol levels and the challenge-activity score (*r* = 0.171), indicating that higher cortisol levels promoted activity among the students (Table 4.).

Another analysis was performed to assess the experienced (cortisol measurement) and estimated stress (KOS score) taking into account demographic variables such as age, gender and place of residence. Detailed results of this analysis are presented in Table 5. Significant correlations were found only between age and cortisol measurement in a low-fidelity simulation setting. It was shown that the mean pre-simulation cortisol level in participants < 25 years of age was *M* = 100.83 (*SD* = 34.11). There was a statistically significant increase (*p* < 0.000) in cortisol levels to *M* = 127.56 (*SD* = 34.61) after a low-fidelity session. The mean pre-simulation cortisol level was *M* = 87.16 (*SD* = 40.20) in students ≥ 25 years of age. There was a statistically significant increase (*p* < 0.000) in cortisol levels to *M* = 113.28 (*SD* = 43.96) after a low-fidelity session also in this age group. For the high-fidelity method, no statistically significant differences were found in cortisol levels in younger and older participants.

In conclusion, both participants ≤ and >25 years of age showed increased levels of stress after low and high-fidelity simulation; however, statistically significantly higher stress levels were observed only for the low-fidelity method.

## 5. Discussion

Humans are exposed to stress in all areas of their activity. Hans Selye was the first to propose the biological concept of stress [32]. He distinguished between the concepts of positive and negative stress. Distress (negative stress) is a state of deprivation or overload that leads to disorganization. Eustress (positive stress), on the other hand, leads to personality development despite temporary discomfort. In the light of recent reports, stress reactions are definitely considered as specific, i.e., dependent on the nature of the stressor and individual characteristics of a given person. The period of studies is undoubtedly associated with significant exposure to stress related to entering adulthood, becoming independent, and building new relationships. The first year of university is particularly stressful due to the accumulation of multiple stressors arising from separation from family, adjusting to college demands, and facing confrontation as a student [33,34,35].

It has repeatedly been shown that nursing students are particularly vulnerable to stress and anxiety reactions due to the nature of the faculty itself, the specificity of classes, multiple demands, and the complexity of expectations. These have a detrimental effect on the sense of control and self-efficacy, and these, after all, are particularly expected of these students upon completion of their education [34,36,37].

McGuire and Lorenz reviewed 2009–2016 publications to analyze data on the levels of stress experienced by medical students and practitioners during simulation and the impact of stress on performance [38]. Student stress levels in simulated conditions were also measured by cortisol levels. The authors often measured cortisol immediately before and immediately after the simulation. In all the studies reviewed, cortisol levels increased after simulation, providing strong evidence that simulation can induce stress. The authors’ findings suggest that cortisol is an important measure of stress in simulation. Of the 17 publications meeting the criteria for inclusion in the review, findings from six articles indicated impaired performance when stress was elevated, with lower performance scores observed in groups of highly stressed students. However, the evidence is not conclusive as to whether elevated stress during simulation improves performance. In contrast, our results showed a very weak statistically significant positive correlation between pre-simulation cortisol levels and the challenge-activity score (r = 0.171) for the high-fidelity method, indicating that a slight increase in cortisol levels may contribute to student activity.

In contrast, Kang and Min conducted a qualitative study to verify students’ perceptions of psychological safety in high-fidelity nursing simulation and standardized patient scenarios [39]. The results indicated that students experienced anxiety, concern, and even fear during and after the nursing simulation, emotions that go beyond the concept of anxiety. In this study, students reported that their increased stress levels largely resulted from the fact that the sessions were recorded by other students and then watched during debriefing. The awareness that their actions were being watched by the teacher and other students led to blockage, increased perceived anxiety, and could consequently lead to more mistakes being made [34]. Perhaps the groups of practicing students should be reduced, and the recording of the sessions should be discussed only in a small group of those directly involved in performing the simulation scenario, which will increase the sense of intimacy and thus reduce stress levels.

Significant psychological stress can impair the learning process and affect the physical and mental health of students [40] therefore, stress monitoring is needed to verify whether the stress experienced during classes is physiological and arises from the learning process or whether it is destructive. Systematic assessment of stress and anxiety among students will allow university staff to quickly identify the problem and take appropriate measures if necessary. Evidence suggests that stress among students can be reduced through various interventions [34].

Our study indicates that low-fidelity simulation induced more physiological and subjectively perceived stress than high-fidelity sessions. Low-fidelity sessions employed simple simulators, e.g., a vascular access trainer or BLS trainer torso, etc. The training on the simulators is usually done individually, with each student practicing on their own and then passing the acquired practical skills. High-fidelity simulation, on the other hand, uses advanced mannequins with a number of functions that imitate the parameters of the human body. Learning with high-fidelity simulators is mostly scenario-based and performed by a team/group of students, usually after mastering basic nursing skills in a low-fidelity simulation setting [41]. Thus, students may feel more confident having already mastered the fundamental skills, having the support of their groupmates, and knowing that they share responsibility for the success or failure of the scenario they are playing out.

Spies and Botma showed that students should be thoroughly acquainted with simulation practice and know in advance what will be expected of them when participating in scenarios [42]. The authors suggest that the sessions should start with less complex scenarios before moving on to more complex ones, which will prevent cognitive overload. Additionally, they point out that failures should not be punished during the debriefing and discussion so that the students could get more engaged and motivated to perform better in subsequent simulation training sessions [42].

In the context of stress, it is impossible to ignore the alarming statistics that show a systematic increase in mental disorders and emotional difficulties among students. Norwegian research has shown that the percentage of students with mental disorders doubled (from 16% to 29%) between 2010 and 2018. This is accompanied by loneliness in 20% of students [43].

It is difficult to compare the analysis of self-reported dispositional stress measured with KOS-B before and after low and high-fidelity simulation sessions with similar studies due to the lack of analogous empirical literature reports. However, our study sheds some light on the issue of university staff education. University teachers working with students in a simulation setting should be properly trained in conducting this type of classes [44,45,46]. This includes the ability to develop scenarios that consider the outcomes of learning, observation, and appropriate responses. Lecturers become trainers and instructors of simulation, which requires them to conduct sessions in such a way that they do not cause destructive stress or negative emotions.

Our study has shown that participation in low and high-fidelity simulation training induces physiological stress reactions that contribute positively to educational outcomes from the psychosocial point of view. They promote attitudes and behaviors that are important for the future nursing practice, and therefore bear the hallmarks of positive, mobilizing eustress.

This study has also some limitations. First, a variable regarding previous experiences might be a potential bias of this study as most of the population is professionally active and have experience with stressful situation. Second, the group was small and a larger sample size in future research may improve the fit of the model, offering more robust results. Despite these limitations, the findings from this study provide a rich source of data.

## 6. Conclusions

Stress levels in the education of nursing students using low and high-fidelity simulation methods can be a factor to reduce the sense of threat and activate professional performance of tasks. Low and high-fidelity simulation sessions do not generate destructive levels of stress. Doubts may arise from the constant testing of the objective (blood) and subjective (KOS-B) levels of stress in the education process.

Nevertheless, constant monitoring of students’ perceived stress levels associated with different types of teaching techniques is needed so that lecturers are aware of which teaching methods most effectively support acquiring new skills by students and while at the same time are student-friendly from the point of view of maintaining their mental well-being. Other methods of testing the level of cortisol (from saliva or hair) could help.

## Figures and Tables

**Table 1:** Demographic characteristics of the study group.

			Age				Education				Place of Residence			
							Secondary		Higher		R	U < 100	U > 100	SU
	N	%	Min–Max	M	MD	*SD*	N	%	N	%	%	%	%	%
total	146	100	19–50	31.83	31.00	7.76	83	56.8	63	43.2	47.9	28.1	21.9	2.1
women	124	84.9	19–50	32.27	32	7.96	71	57.3	53	42.7				
men	22	15.1	20–42	29.63	28.50	6.09	12	54.5	10	45.5				

R—rural; U—urban, SU—suburban.

**Table 2:** Reference range for serum cortisol in the laboratory where the study material was analyzed.

			95% Reference Range	
Type of Sample	Sampling	n	µg/dL	Nmol/dL
serum	Before 10 a.m.	150	3.7–19.4	102.1–535.2
serum	After 5:00 p.m.	150	2.9–17.3	80.0–477.3

**Table 3:** Descriptive statistics of study variables.

	M	MD	*SD*	Skewness	Kurtosis	λ	*p*
KOS_GEN_before	22.22	21.00	6.07	0.113	0.824	0.107	<0.001
KOS_GEN_after	26.38	28.00	6.90	−0.513	−0.148	0.104	<0.001
KOS_THREAT_before	3.29	3.00	3.18	1.364	1.443	0.214	<0.001
KOS_THREAT_after	3.29	3.00	3.18	1.364	1.443	0.214	<0.001
KOS_CHAL_ACT_before	8.23	8.00	2.35	−0.025	0.674	0.104	<0.001
KOS_CHAL_ACT_after	8.23	8.00	2.35	−0.025	0.674	0.104	<0.001
KOS_CHAL_PAS_before	9.47	9.00	2.65	−0.126	0.789	0.087	0.002
KOS_CHAL_PAS_after	9.47	9.00	2.65	−0.126	0.789	0.097	0.002
KOS_HARM/LOSS_before	1.23	1.00	1.46	1.321	1.342	0.228	<0.001
KOS_HARM/LOSS_after	1.23	1.00	1.46	1.321	1.342	0.228	<0.001
Cortisol_1_before	90.53	82.00	39.12	1.186	1.701	0.132	<0.001
Cortisol_1_after	116.80	112.5	42.19	1.304	2.286	0.062	0.200 *
Cortisol_2_before	112.75	106.00	42.44	1.008	1.803	0.106	<0.001
Cortisol_2_after	117.57	121.50	44.80	0.292	0.180	0.051	0.200 *

KOS_GEN—KOS general; KOS_THREAT—KOS threat; KOS_CHAL_ACT—KOS challenge activities; KOS_CHAL_PAS—KOS challenge passive; KOS_HARM/LOSS—KOS harm/loss; *—statistical significance.

**Table 4:** Self-reported dispositional stress of respondents before and after low-fidelity simulation sessions.

		Threat	Challenge Activity	Challenge Passivity	Harm/Loss
		Measurement 1	Measurement 1	Measurement 1	Measurement 1
cort. sampling 1 BEFORE	Spearman’s rho	−0.153	0.014	−0.129	−0.117
	*p*	0.065	0.866	0.122	0.160
cort. sampling 1 AFTER.	Spearman’s rho	−0.194 *	−0.006	−0.046	−0.079
	*p*	0.019 *	0.940	0.584	0.342
		**Measurement 2**	**Measurement 2**	**Measurement 2**	**Measurement 2**
cort. sampling 2 BEFORE	Spearman’s rho	0.011	0.17 *	0.056	0.011
	*p*	0.895	0.039 *	0.502	0.900
cort. sampling 2 AFTER	Spearman’s rho	−0.126	0.036	−0.001	0.017
	*p*	0.130	0.668	0.988	0.836

cort. sampling—cortisol sampling; 1 measurement—low-fidelity simulation; 2 measurement—high fidelity simulation. *—statistical significance.

**Table 5:** The rho-Spearman correlation analysis between age and the experienced and perceived stress.

		Cortisol 1_before	Cortisol 1_after	Cortisol 2_before	Cortisol 2_after	KOS_G1	KOS_G2	KOS_D_1	KOS_D_2	KOS_C_A_1	KOS_C_A_2	KOS_C_P_1	KOS_C_P_2	KOS_H/L	KOS_H/L
age	rho	−0.212 *	−0.214 *	−0.149	−0.085	0.152	0.022	0.077	0.077	0.090	0.090	0.158	0.158	0.034	0.034
	*p*	0.010 *	0.010 *	0.073	0.305	0.067	0.795	0.354	0.354	0.279	0.279	0.057	0.057	0.680	0.680

KOS_D—KOS_danger; KOS_C_A—KOS_challenge_activity; KOS_C_P—KOS_challenge_pasivity; KOS_H/L—KOS_harm/loss, *—statistical significance.

## Data Availability

Not applicable.
